# Impact of Cytological Sampling on EGFR Mutation Testing in Stage III-IV Lung Adenocarcinoma

**DOI:** 10.1155/2017/9614938

**Published:** 2017-03-07

**Authors:** Rhian Siân Davies, Christian Smith, Gwenllian Edwards, Rachel Butler, Diane Parry, Jason Francis Lester

**Affiliations:** ^1^Velindre Cancer Centre, Cardiff, UK; ^2^Institute of Medical Genetics, University Hospital of Wales, Cardiff, UK; ^3^University Hospital Llandough, Cardiff, UK

## Abstract

*Objectives*. There have been advances in the identification and understanding of molecular subsets of lung cancer, defined by specific oncogenic aberrations. A number of actionable genetic alterations have been identified, such as the epidermal growth factor receptor (EGFR) mutation. We aimed to establish the reasons why patients were not undergoing EGFR mutation testing at the time of histological diagnosis.* Methods*. The records of 70 patients with advanced adenocarcinoma of the lung managed through a single multidisciplinary team at a single institution were reviewed. Data were collected on method of tumour sample collection, whether this was sent for EGFR testing, and the result.* Results*. Seventy patients were identified. In 21/25 (84%) cases, cytological sampling was sufficient for EGFR mutation analysis, compared with 40/45 (89%) cases with histological sampling. EGFR mutation testing was not carried out in 22/70 (31.4%) patients. There was insufficient tumour sample for EGFR testing in 9/22 (40.9%) patients. Other reasons for not testing included poor patient fitness and problems in the diagnostic pathway.* Conclusions*. In this series, cytological tumour sampling was not the predominant reason why cancers failed to have EGFR mutation status established.

## 1. Introduction

Lung cancer represents a significant health problem. In Europe in 2012, there were an estimated 410,000 new cases diagnosed, and most patients present with advanced stage, incurable disease [1].

In recent years, there have been advances in the identification and understanding of molecular subsets of lung cancer, defined by specific oncogenic aberrations [2]. There are a number of actionable genetic alterations that have been identified.

Oral tyrosine kinase inhibitors (TKIs) have been shown to prolong progression-free survival in patients with advanced NSCLC harbouring a sensitising EGFR mutation compared with doublet chemotherapy in a number of clinical trials [3–5]. In addition, recent analysis of data from the LUX-LUNG 3 and LUX-LUNG 6 trials has shown that in patients with exon 19 deletions (the commonest sensitising EGFR mutation), afatinib significantly prolongs overall survival in the first-line setting in stage IIIB-IV NSCLC when compared with doublet chemotherapy [3, 4]. The EML4-ALK translocation protein is found in approximately 3–7% of adenocarcinomas and 2–5% of NSCLC overall [2]. Crizotinib is an oral small molecule TKI which specifically targets ALK, MET, and ROS1. The PROFILE 1007 trial demonstrated a significantly longer progression-free survival with Crizotinib compared with chemotherapy in the second-line setting for patients with advanced ALK positive NSCLC [6]. In addition, several other genetic alterations have been identified. These include ROS1, AKT, BRAF, FGFR, MET, MEK1, PTEN RET, PIK3CA, KRAS, and HER2 [2]. Treatments that target these alterations are currently undergoing testing in clinical trials and may have therapeutic application in the near future. Therefore, it is imperative that adequate samples are acquired for molecular profiling to ensure patients receive optimum treatment and to facilitate patients entering clinical trials.

There has been a drive in recent years to use minimally invasive techniques to obtain sufficient tumour sample for diagnosis in patients with advanced NSCLC. The National Institute for Health and Clinical Excellence (NICE) recommend choosing the investigation which gives the most information about diagnosis and staging with the least risk to the patient [7]. In light of this, techniques such as endoscopic bronchial ultrasound- (EBUS-) guided biopsy, bronchoscopy with fine needle aspiration (FNA), and ultrasound-guided biopsy of supraclavicular neck nodes have become more commonly used. These techniques provide cytological samples for testing. We hypothesised that this shift in diagnostic approach may have led to a proportion of cases where molecular analysis was not possible due to insufficient cancer DNA in the tumour samples provided. To test this hypothesis, as well as investigating other reasons why patients' tumour samples were not tested, we looked at the records of 70 consecutive patients with advanced adenocarcinoma of the lung managed through a single multidisciplinary team (MDT) in SE Wales.

## 2. Materials and Methods

All patients diagnosed and treated through a single lung cancer MDT (at a single institution) between January 1, 2012, and January 1, 2014, were identified using Cansic, an electronic patient database record of all cancer patients in Wales. Patients with stage III or IV NSCLC and a histological or cytological diagnosis of adenocarcinoma were included in the analysis. Squamous cell carcinoma cases were excluded; at the time of the study there were no identified molecular subsets that would benefit from a licensed targeted therapy, so samples were not routinely tested. Data were collected based on patient sex, date of birth, age at diagnosis, stage at diagnosis, method of tumour sample collection, whether the sample was sent for EGFR mutation testing, and the results of testing. EGFR mutation analysis was carried out in the All Wales Genetics Laboratory, University Hospital of Wales. In patients where no EGFR mutation testing was performed, the reason for this was recorded. No patients were tested for the ALK translocation, as at the time of the study, no ALK-targeting therapies were routinely funded.

## 3. Results

In total, 70 patients who fulfilled the criteria were identified and all were included in the analysis. There were 42 females and 28 males. The median age was 67 years (range 42–84 years). In total, 12/70 (17%) patients had stage IIIA disease, 5/70 (7%) had stage IIIB, and 53/70 (76%) had stage IV disease. A histological sampling technique was used in 45/70 (64%) patients, and a cytological sampling technique was used in 25/70 (36%) patients.

In total, 48/70 (68.6%) patients in this study diagnosed with stage III/IV adenocarcinoma of the lung had EGFR mutation testing. EGFR mutation testing was not carried out in 22/70 (31.4%) patients (Table 1). In 13/22 (59.1%) of cases, there was sufficient sample for EGFR mutation analysis, but testing was not carried out, in 10/22 (45.5%) patients there was no sample sent and no documented reason, 2/22 (9.1%) patients were not fit for treatment, and 1/22 (4.5%) patient declined treatment. In the remaining 9/22 (41%) patients, there was insufficient sample for EGFR mutation testing.

Therefore, in total, sufficient tumour sample was acquired for EGFR mutation analysis in 61/70 (87%) patients. In 21/25 (84%) cases, sufficient sample was acquired for EGFR mutation analysis after a cytological sampling technique, compared with 40/45 (89%) with a histological sampling technique. There was insufficient sample for analysis in 9/70 (13%) patients. Of these, 3 patients had a sample obtained from a CT guided biopsy, 2 had a bronchoscopic biopsy, 1 EBUS FNA of a node, 1 bronchial washing, 1 bone metastasis biopsy, and 1 sample was a pleural fluid sample. The diagnostic yield according to sampling method is shown in Table 2.

Wild type EGFR was identified in 41/48 (85.4%) of patients tested. 7/48 (14.6%) tested patients had a sensitising EGFR mutation.

## 4. Discussion

This is a retrospective real world study looking at the proportion of patients with newly diagnosed stage III/IV adenocarcinoma of the lung whose diagnostic tumour sample underwent successful EGFR mutation analysis. Treatment decisions are now based not only on histological subtype but increasingly on molecular profiling, most importantly EGFR mutation status. The LUX-Lung 3 and 6 trials have shown a significant overall survival benefit in the first-line setting with the EGFR TKI afatinib in patients with the EGFR exon 19 deletion, highlighting the importance of adequate tumour sampling to allow genetic analysis to be undertaken [3, 4].

In this cohort, 22/70 (31.4%) patients did not undergo EGFR mutation testing. Our hypothesis was that this may have been due to cytological tumour sampling resulting in less tumour DNA for testing. In fact, results showed that the percentage of successful EGFR tests was similar in the cytological (21/25, 84%) and histological (40/45, 88.9%) sample groups. The similarity in successful mutation analysis between the two groups effectively disproved the hypothesis that the dominant reason for the lower than expected rate of mutation analysis was a result of insufficient sample from a cytological diagnostic techniques. This observation is borne out in other published studies looking at methods of acquiring sample in patients with lung cancer [8]. A study by José et al. retrospectively assessed the methods used to obtain diagnostic samples in 328 consecutive patients diagnosed with lung cancer between 2007 and 2011. They found that there had been a reduction in the number of standard bronchoscopies and mediastinoscopies carried out and a significant increase in EBUS-transbronchial needle aspiration (TBNA) [9]. Jurado et al. reported on 56 patients with adenocarcinoma, who underwent EBUS FNA, and showed this is effective for molecular testing, providing sufficient sample for testing in 82% of patients in the study [10]. This is comparable to the rate seen in our series using cytological diagnostic techniques. A recent systematic review by Ellison et al. identified 33 studies reporting the use of cytology for EGFR mutation testing, including FNA samples obtained under CT guidance, endoscopic ultrasound (EUS), and EBUS, which concluded that these samples can be successfully assessed for EGFR mutations using various techniques such as direct sequencing, real-time PCR, and COLD-PCR [11]. Albanna et al. compared the yield for 702 diagnostic procedures for pathological classification, of which 269 samples were also sent for EGFR mutation analysis. They concluded that radiologically guided TBNA provided a high yield for molecular analysis, but unguided TBNA was of low utility, demonstrating the importance of using radiological guidance when undertaking TBNA [12].

It is clear that in our cohort of patients the most significant reason for samples not being tested for EGFR mutations was failure in the process around requesting EGFR testing. One way of minimising delays and simplifying the process to ensure all relevant samples are tested is by introducing protocol driven EGFR testing. In other words all samples are sent for EGFR mutation testing by the reporting histopathologist irrespective of histologic subtype, disease stage, treatment intent, or potential clinical trial participation. In this study, patients were selected for EGFR mutation testing on the basis of adenocarcinoma histology and advanced disease. In clinical practice, our selection criteria for EGFR mutation testing are very similar; all patients with nonsquamous NSCLC and advanced incurable disease are tested. We do not routinely test patients with squamous cell carcinoma as mutation rates are reported to be very low. In addition, we do not test patients with localised or locally advanced disease undergoing potentially curative treatment because mutation status is highly unlikely to change initial management. Clinical trials often require central review of pathology or additional sample for nonstandard tests. Diagnostic samples are often scanty and a decision may have to be made whether to use the specimen for immunohistochemical testing, EGFR mutation testing, or central review at a clinical trials laboratory. For these reasons, the decision whether to EGFR mutation test or not is made at the MDT for a significant proportion of patients.

In ten patients, biopsy samples were not sent for EGFR mutation analysis. This represents a significant proportion of our cohort. At the time of the study, there was no method of tracking a sample through the process from acquisition to EGFR mutation testing. In addition, the EGFR mutation result is not automatically incorporated into the biopsy pathology report. In these cases, it was not possible to determine exactly where the pathway had broken down, and we are therefore unable to comment on why EGFR testing was not carried out. In our opinion, a possible explanation is that there were a proportion of cases where an assumption had been made that the sample taken was insufficient for testing. There is no defined standard for minimum sample requirement for EGFR mutation analysis. Guidelines from the College of American Pathologists state that each laboratory should establish the minimum proportion and number of cancer cells needed for mutation detection during validation and that pathologists should determine the adequacy of a specimen by assessing cancer cell content, DNA quantity, and quality [13]. Consequently, these decisions are inevitably subjective and will vary between clinicians and laboratories. It is known that EGFR mutation analysis can be carried out on very small samples. A recent study showed that successful mutation analysis could be carried out on samples as small as 0.12 mm^2^, containing less than 200 cells [14]. Our practice now is to send even very small samples for mutation analysis.

In two cases, EGFR mutation analysis was not carried out due to the patients' poor performance status (PS), in both cases the World Health Organisation PS (WHO PS) was 3, and the patient was deemed not fit for treatment. There is evidence that very poor PS patients with an actionable EGFR mutation do benefit from an EGFR TKI even if they are not fit enough for treatment with chemotherapy [15]. It is now standard practice for us to test patients with poor PS for EGFR mutations, as the “Lazarus effect” in mutation positive patients treated with EGFR TKI is well recognized [16].

As our understanding of the molecular mechanisms underlying tumour pathogenesis increases, more targeted therapies are becoming available for this group of patients. Although molecular technologies will improve through gene panel analysis, the requirement for sufficient sample for increasingly complex molecular analyses will become more important for effective treatment decisions. In the near future, the use of circulating tumour DNA (ctDNA) as a diagnostic sampling method may improve some of these practical issues. ctDNA is shed from tumours into peripheral blood during apoptosis, providing easy accessibility despite minimal tumour DNA samples. The use of ctDNA allows diagnostic analysis of targeted molecular biomarkers such as EGFR without the requirement for a sample. This therefore enables access to testing for those lung cancer patients who do not have sufficient sample or have not been sampled at all. However, this technology relies upon careful sample handling, as ctDNA is highly degradable. The yield of ctDNA for diagnostic analysis is limited and sufficient for EGFR but may prove challenging with the requirement for gene panel testing. The introduction of digital methodologies has now significantly improved the sensitivity of analysis such that false negatives are no longer a concern. The benefits of ctDNA include less invasive sampling, with the option of easily repeated samples, and this may be extended to sampling for the detection of resistance mutations such as EGFR p.T790M. ctDNA also avoids issues of sample heterogeneity which are associated with small biopsies.

## 5. Conclusion

This study was carried out because of concerns around a low EGFR mutation testing rate in patients with advanced NSCLC potentially suitable for targeted therapy. Insufficient sample acquisition was not the most significant reason for the low testing rate seen; cytological sampling was almost as effective as histological methods at acquiring adequate sample for analysis. A greater problem was the pathway for requesting EGFR mutation analysis, which meant that appropriate patients were not being tested for the EGFR mutation despite adequate sample and were being denied the opportunity to have potentially effective treatment. We recommend that all lung cancer MDTs review their local EGFR mutation testing practice to ensure robust systems are in place and patients are given every opportunity to receive optimum treatment.

## Figures and Tables

**Table 1:** Reasons why EGFR testing was not done.

	*N*	%
Insufficient sample for EGFR mutation testing	9	40.9
(i) Histological tumour sampling	*(6)*	*(27.3)*
(ii) Cytological tumour sampling	*(3)*	*(13.6)*
No sample sent (no documented reason)	10	45.5
Deemed not fit for treatment	2	9.1
Declined treatment	1	4.5

**Table 2:** Source of sample for diagnosis.

		Sent for EGFR mutation testing		Successful mutation analysis	
	*N*	*N*	%	*N*	%
*Histological*	*45*	*37*	*82*	*30*	*81*
CT guided lung biopsy	17	14	82	11	79
Bronchoscopic biopsy	12	9	75	8	89
CT guided bone biopsy	3	3	100	3	100
Wedge resection	2	2	100	0	0
Excision biopsy neck node	2	2	100	2	100
Biopsy of metastasis	2	2	100	2	100
Lobectomy	1	1	100	1	100
VATS biopsy lung	1	0	0	0	0
Core biopsy node	1	1	100	1	100
Bone biopsy	3	2	67	1	33
Endoscopic biopsy of oesophagus	1	1	100	1	100
*Cytological *	*25*	*22*	*88*	*20*	*91*
Pleural fluid	11	10	91	9	90
EBUS TBNA node	9	8	89	8	100
Bronchial washings	2	1	50	0	0
CT guided FNA lung	1	1	100	1	100
EUS FNA node	1	1	100	1	100
FNA node	1	1	100	1	100
